# 1-Benzyl-2-(4-chloro­phen­yl)-4,5-di­phenyl-1*H*-imidazole

**DOI:** 10.1107/S1600536808016802

**Published:** 2008-06-07

**Authors:** Mahmood Ghoranneviss, Ghodsi Mohammadi Ziarani, Alireza Abbasi, Mohammad Reza Hantehzadeh, Zahra Farahani

**Affiliations:** a Plasma Physics Research Center, Science & Reseach Campus, Islamic Azad University, Tehran, Iran; b Department of Chemistry, University of Alzahra, Tehran, Iran; c School of Chemistry, University College of Science, University of Tehran, Tehran, Iran

## Abstract

The mol­ecular conformation of the title compound, C_28_H_21_ClN_2_, is stabilized by an intra­molecular C—H⋯N hydrogen bond. It has many pharmacological properties, such as being an inhibitor of P38 MAP Kinase, and can play an important role in biochemical processes.

## Related literature

For related structures and properties, see: Balalaie *et al.* (2003 ▶); Nagarapu *et al.* (2007 ▶); Kidwai *et al.* (2007 ▶).
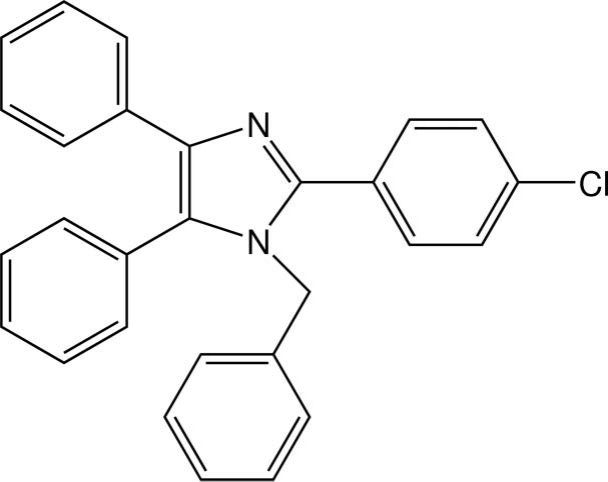


## Experimental

### 

#### Crystal data

C_28_H_21_ClN_2_
*M* *_r_* = 420.92
Triclinic, 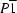

*a* = 7.4880 (11) Å
*b* = 9.2711 (16) Å
*c* = 16.049 (3) Å
α = 87.169 (13)°
β = 76.704 (12)°
γ = 87.842 (13)°
*V* = 1082.6 (3) Å^3^
*Z* = 2
Mo *K*α radiation
μ = 0.19 mm^−1^
*T* = 290 (2) K
0.3 × 0.2 × 0.2 mm

#### Data collection

STOE IPDS-II diffractometer
Absorption correction: none
8769 measured reflections
4246 independent reflections
2814 reflections with *I* > 2σ(*I*)
*R*_int_ = 0.034

#### Refinement

*R*[*F* ^2^ > 2σ(*F* ^2^)] = 0.041
*wR*(*F* ^2^) = 0.090
*S* = 0.94
4246 reflections
288 parameters
H atoms treated by a mixture of independent and constrained refinement
Δρ_max_ = 0.13 e Å^−3^
Δρ_min_ = −0.20 e Å^−3^

### 

Data collection: *X-AREA* (Stoe & Cie, 1997 ▶); cell refinement: *X-AREA*; data reduction: *X-AREA*; program(s) used to solve structure: *SHELXS97* (Sheldrick, 2008 ▶); program(s) used to refine structure: *SHELXL97* (Sheldrick, 2008 ▶); molecular graphics: *DIAMOND* (Brandenburg, 2001 ▶); software used to prepare material for publication: *SHELXL97*.

## Supplementary Material

Crystal structure: contains datablocks I, 1. DOI: 10.1107/S1600536808016802/bt2715sup1.cif

Structure factors: contains datablocks I. DOI: 10.1107/S1600536808016802/bt2715Isup2.hkl

Additional supplementary materials:  crystallographic information; 3D view; checkCIF report

## Figures and Tables

**Table 1:** Hydrogen-bond geometry (Å, °)

*D*—H⋯*A*	*D*—H	H⋯*A*	*D*⋯*A*	*D*—H⋯*A*
C3—H3⋯N1	0.93	2.56	2.874 (2)	100

**Fig. 1.**
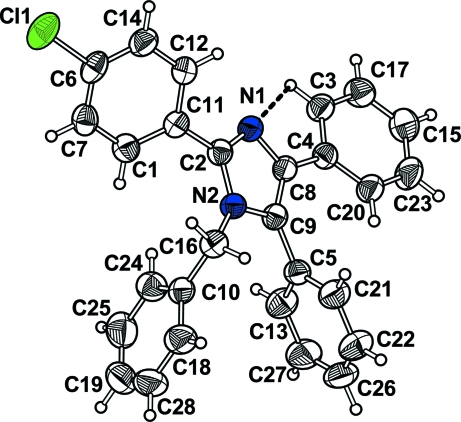
Molecular structure of (I), with 50% probability displacement ellipsoids. H atoms are shown as circles of arbitrary radii.

## supplementary crystallographic information

### Comment

The synthesis, reactions and biological properties of substituted imidazole constitutes a significant part of modern heterocyclic chemistry. Compounds containing an imidazole ring system have many pharmacological properties and play important roles in biochemical processes. Many of substituted diaryl imidazoles are known as inhibitors of P38 MAP Kinase (Balalaie, *et al.*, 2003; Nagarapu, *et al.*, 2007; Kidwai, *et al.*, 2007).

The molecular structure of the title compound and the atom-numbering scheme are shown in Fig. 1. The phenyl rings attached at C2, N2, C9 and C8 enclose dihedral angles of 40.74 (8)°,  85.60 (7)°,    77.10 (6)°, 16.55 (11)°, respectively, with the imidazole ring. An intramolecular hydrogen bond stabilises the molecular conformation. Dipole-dipole and van der Waals interactions are   effective in the molecular packing.

### Experimental

A mixture of benzil (2.5 mmol), 4-chlorobenzaldehyde (2.5 mmol), benzylamine (2.5 mmol), ammonium acetate (5 mmol) and activated SBA-sulfonic acid (0.02 g) was heated at 140°C for 6 minutes. The progress of reaction was monitored by TLC. After cooling to room temperature, the mixture was dissolved in hot ethylacetate and the catalyst was removed by filtration. The filtrate was left for crystallization.

### Refinement

Aromatic H atoms were placed in calculated positions (C—H = 0.93 Å) and constrained to ride on their parent atoms, with *U*_iso_(H) = 1.2Ueq(C). Methylene   H atoms were located in difference density maps and their coordinates and isotropic displacement parameters were refined freely.

### Crystal data

**Table:**

C_28_H_21_ClN_2_	*Z* = 2
*M**_r_* = 420.92	*F*_000_ = 440
Triclinic, *P*1	*D*_x_ = 1.291 Mg m^−^^3^
Hall symbol: -P 1	Mo *K*α radiation λ = 0.71073 Å
*a* = 7.4880 (11) Å	Cell parameters from 6987 reflections
*b* = 9.2711 (16) Å	θ = 1.2–29.8º
*c* = 16.049 (3) Å	µ = 0.19 mm^−^^1^
α = 87.169 (13)º	*T* = 290 (2) K
β = 76.704 (12)º	Block, colorless
γ = 87.842 (13)º	0.3 × 0.2 × 0.2 mm
*V* = 1082.6 (3) Å^3^	

### Data collection

**Table:**

STOE IPDS-II diffractometer	2814 reflections with *I* > 2σ(*I*)
Radiation source: fine-focus sealed tube	*R*_int_ = 0.034
Monochromator: graphite	θ_max_ = 26.0º
*T* = 290(2) K	θ_min_ = 2.2º
Area detector – phi oscillation scans	*h* = −9→9
Absorption correction: none	*k* = −11→10
8769 measured reflections	*l* = −19→19
4246 independent reflections	

### Refinement

**Table:**

Refinement on *F*^2^	Secondary atom site location: difference Fourier map
Least-squares matrix: full	Hydrogen site location: inferred from neighbouring sites
*R*[*F*^2^ > 2σ(*F*^2^)] = 0.041	H atoms treated by a mixture of independent and constrained refinement
*wR*(*F*^2^) = 0.090	*w* = 1/[σ^2^(*F*_o_^2^) + (0.0457*P*)^2^] where *P* = (*F*_o_^2^ + 2*F*_c_^2^)/3
*S* = 0.94	(Δ/σ)_max_ < 0.001
4246 reflections	Δρ_max_ = 0.13 e Å^−^^3^
288 parameters	Δρ_min_ = −0.20 e Å^−^^3^
Primary atom site location: structure-invariant direct methods	Extinction correction: none

### Special details

**Table:**

Geometry. All e.s.d.'s (except the e.s.d. in the dihedral angle between two l.s. planes) are estimated using the full covariance matrix. The cell e.s.d.'s are taken into account individually in the estimation of e.s.d.'s in distances, angles and torsion angles; correlations between e.s.d.'s in cell parameters are only used when they are defined by crystal symmetry. An approximate (isotropic) treatment of cell e.s.d.'s is used for estimating e.s.d.'s involving l.s. planes.
Refinement. Refinement of *F*^2^ against ALL reflections. The weighted *R*-factor *wR* and goodness of fit *S* are based on *F*^2^, conventional *R*-factors *R* are based on *F*, with *F* set to zero for negative *F*^2^. The threshold expression of *F*^2^ > σ(*F*^2^) is used only for calculating *R*-factors(gt) *etc*. and is not relevant to the choice of reflections for refinement. *R*-factors based on *F*^2^ are statistically about twice as large as those based on *F*, and *R*- factors based on ALL data will be even larger.

### Fractional atomic coordinates and isotropic or equivalent isotropic displacement parameters (Å^2^)

**Table:**

	*x*	*y*	*z*	*U*_iso_*/*U*_eq_	
Cl1	0.17061 (7)	0.52946 (7)	0.32233 (4)	0.0741 (2)	
N1	0.60096 (17)	0.82368 (15)	0.59681 (9)	0.0412 (3)	
N2	0.36337 (17)	0.74456 (15)	0.69583 (8)	0.0403 (3)	
C1	0.2919 (2)	0.56492 (19)	0.54889 (12)	0.0456 (4)	
H1	0.2804	0.5096	0.5997	0.055*	
C2	0.4446 (2)	0.75675 (17)	0.61039 (10)	0.0393 (4)	
C3	0.9378 (2)	0.9430 (2)	0.61528 (12)	0.0476 (4)	
H3	0.9370	0.8959	0.5656	0.057*	
C4	0.7878 (2)	0.93196 (18)	0.68467 (11)	0.0413 (4)	
C5	0.4409 (2)	0.80553 (19)	0.83352 (11)	0.0419 (4)	
C6	0.2487 (2)	0.5939 (2)	0.40748 (12)	0.0492 (5)	
C7	0.2308 (2)	0.5117 (2)	0.48201 (12)	0.0491 (4)	
H7	0.1784	0.4213	0.4874	0.059*	
C8	0.6244 (2)	0.85566 (17)	0.67670 (10)	0.0391 (4)	
C9	0.4790 (2)	0.80756 (18)	0.73880 (11)	0.0398 (4)	
C10	0.1795 (2)	0.55784 (19)	0.79077 (11)	0.0435 (4)	
C11	0.3703 (2)	0.69955 (18)	0.54181 (11)	0.0399 (4)	
C12	0.3880 (2)	0.77975 (19)	0.46497 (11)	0.0475 (4)	
H12	0.4418	0.8696	0.4587	0.057*	
C13	0.5348 (3)	0.7078 (2)	0.87682 (12)	0.0567 (5)	
H13	0.6178	0.6416	0.8463	0.068*	
C14	0.3263 (3)	0.7273 (2)	0.39788 (12)	0.0530 (5)	
H14	0.3373	0.7817	0.3468	0.064*	
C15	1.0919 (3)	1.0925 (2)	0.69172 (14)	0.0607 (5)	
H15	1.1921	1.1472	0.6939	0.073*	
C16	0.1802 (2)	0.6943 (2)	0.73629 (12)	0.0440 (4)	
H16A	0.114 (2)	0.6806 (18)	0.6900 (11)	0.046 (5)*	
H16B	0.114 (2)	0.7692 (19)	0.7710 (11)	0.046 (5)*	
C17	1.0877 (2)	1.0226 (2)	0.61875 (13)	0.0550 (5)	
H17	1.1863	1.0290	0.5715	0.066*	
C18	0.0320 (3)	0.5323 (2)	0.85851 (13)	0.0625 (5)	
H18	−0.0639	0.6003	0.8704	0.075*	
C19	0.1645 (4)	0.3071 (3)	0.89254 (17)	0.0854 (8)	
H19	0.1596	0.2226	0.9267	0.102*	
C20	0.7963 (2)	1.0024 (2)	0.75824 (12)	0.0505 (4)	
H20	0.6988	0.9963	0.8060	0.061*	
C21	0.3165 (3)	0.9005 (2)	0.88092 (12)	0.0577 (5)	
H21	0.2503	0.9662	0.8533	0.069*	
C22	0.2891 (3)	0.8988 (3)	0.96904 (13)	0.0699 (6)	
H22	0.2046	0.9633	1.0003	0.084*	
C23	0.9463 (3)	1.0807 (2)	0.76139 (13)	0.0604 (5)	
H23	0.9495	1.1263	0.8113	0.072*	
C24	0.3199 (3)	0.4554 (2)	0.77506 (13)	0.0557 (5)	
H24	0.4208	0.4706	0.7298	0.067*	
C25	0.3117 (4)	0.3305 (3)	0.82608 (17)	0.0747 (6)	
H25	0.4072	0.2621	0.8150	0.090*	
C26	0.3851 (3)	0.8033 (3)	1.01068 (13)	0.0680 (6)	
H26	0.3676	0.8037	1.0699	0.082*	
C27	0.5065 (3)	0.7074 (3)	0.96484 (14)	0.0688 (6)	
H27	0.5708	0.6412	0.9931	0.083*	
C28	0.0251 (4)	0.4068 (3)	0.90890 (15)	0.0820 (7)	
H28	−0.0755	0.3905	0.9542	0.098*	

### Atomic displacement parameters (Å^2^)

**Table:**

	*U*^11^	*U*^22^	*U*^33^	*U*^12^	*U*^13^	*U*^23^
Cl1	0.0667 (3)	0.1008 (5)	0.0631 (3)	−0.0114 (3)	−0.0247 (3)	−0.0313 (3)
N1	0.0440 (7)	0.0441 (8)	0.0370 (8)	−0.0063 (6)	−0.0110 (6)	−0.0032 (6)
N2	0.0392 (7)	0.0453 (8)	0.0374 (8)	−0.0061 (6)	−0.0101 (6)	−0.0014 (6)
C1	0.0421 (9)	0.0474 (11)	0.0493 (10)	−0.0038 (8)	−0.0142 (8)	−0.0024 (8)
C2	0.0399 (8)	0.0402 (10)	0.0396 (9)	−0.0009 (7)	−0.0126 (7)	−0.0028 (7)
C3	0.0473 (9)	0.0531 (11)	0.0429 (10)	−0.0051 (8)	−0.0102 (8)	−0.0057 (8)
C4	0.0445 (9)	0.0407 (10)	0.0407 (9)	−0.0038 (7)	−0.0136 (7)	−0.0021 (7)
C5	0.0408 (8)	0.0473 (10)	0.0383 (9)	−0.0095 (7)	−0.0096 (7)	0.0000 (8)
C6	0.0400 (9)	0.0621 (13)	0.0497 (11)	0.0020 (8)	−0.0156 (8)	−0.0205 (9)
C7	0.0400 (9)	0.0491 (11)	0.0598 (12)	−0.0063 (8)	−0.0124 (8)	−0.0105 (9)
C8	0.0427 (8)	0.0372 (9)	0.0384 (9)	−0.0002 (7)	−0.0109 (7)	−0.0040 (7)
C9	0.0401 (8)	0.0419 (9)	0.0394 (9)	−0.0012 (7)	−0.0128 (7)	−0.0033 (7)
C10	0.0417 (9)	0.0516 (11)	0.0402 (10)	−0.0117 (8)	−0.0136 (8)	−0.0035 (8)
C11	0.0366 (8)	0.0437 (10)	0.0404 (9)	0.0010 (7)	−0.0100 (7)	−0.0064 (8)
C12	0.0558 (10)	0.0434 (11)	0.0443 (10)	−0.0058 (8)	−0.0122 (8)	−0.0055 (8)
C13	0.0580 (11)	0.0655 (13)	0.0497 (12)	0.0048 (9)	−0.0196 (9)	−0.0046 (10)
C14	0.0636 (11)	0.0563 (12)	0.0419 (10)	0.0011 (9)	−0.0174 (9)	−0.0059 (9)
C15	0.0520 (10)	0.0654 (13)	0.0692 (14)	−0.0171 (9)	−0.0200 (10)	−0.0062 (11)
C16	0.0337 (8)	0.0529 (11)	0.0454 (10)	−0.0023 (8)	−0.0083 (8)	−0.0041 (9)
C17	0.0450 (10)	0.0636 (13)	0.0551 (12)	−0.0105 (9)	−0.0080 (9)	−0.0002 (10)
C18	0.0561 (11)	0.0763 (15)	0.0532 (12)	−0.0186 (10)	−0.0061 (9)	−0.0019 (11)
C19	0.140 (2)	0.0633 (16)	0.0644 (16)	−0.0376 (17)	−0.0452 (17)	0.0139 (13)
C20	0.0515 (10)	0.0560 (12)	0.0443 (10)	−0.0104 (8)	−0.0088 (8)	−0.0082 (9)
C21	0.0628 (11)	0.0599 (13)	0.0483 (11)	0.0059 (10)	−0.0092 (9)	−0.0034 (9)
C22	0.0746 (14)	0.0808 (16)	0.0480 (13)	0.0019 (12)	0.0005 (11)	−0.0138 (11)
C23	0.0634 (12)	0.0680 (13)	0.0554 (12)	−0.0141 (10)	−0.0206 (10)	−0.0166 (10)
C24	0.0565 (11)	0.0577 (13)	0.0543 (12)	−0.0039 (9)	−0.0161 (9)	0.0028 (10)
C25	0.0997 (17)	0.0578 (14)	0.0771 (16)	−0.0012 (12)	−0.0425 (14)	0.0001 (12)
C26	0.0800 (14)	0.0880 (17)	0.0359 (11)	−0.0195 (12)	−0.0106 (10)	0.0004 (11)
C27	0.0821 (14)	0.0792 (16)	0.0513 (13)	−0.0035 (12)	−0.0298 (11)	0.0076 (11)
C28	0.0988 (18)	0.092 (2)	0.0522 (14)	−0.0498 (16)	−0.0062 (12)	0.0070 (13)

### Geometric parameters (Å, °)

**Table:**

Cl1—C6	1.7436 (17)	C13—C27	1.379 (3)
N1—C2	1.314 (2)	C13—H13	0.9300
N1—C8	1.381 (2)	C14—H14	0.9300
N2—C2	1.367 (2)	C15—C17	1.373 (3)
N2—C9	1.385 (2)	C15—C23	1.374 (3)
N2—C16	1.459 (2)	C15—H15	0.9300
C1—C7	1.378 (2)	C16—H16A	1.000 (18)
C1—C11	1.388 (2)	C16—H16B	0.960 (17)
C1—H1	0.9300	C17—H17	0.9300
C2—C11	1.471 (2)	C18—C28	1.379 (3)
C3—C17	1.380 (2)	C18—H18	0.9300
C3—C4	1.391 (2)	C19—C28	1.356 (4)
C3—H3	0.9300	C19—C25	1.362 (4)
C4—C20	1.392 (2)	C19—H19	0.9300
C4—C8	1.470 (2)	C20—C23	1.372 (3)
C5—C21	1.378 (2)	C20—H20	0.9300
C5—C13	1.383 (3)	C21—C22	1.381 (3)
C5—C9	1.480 (2)	C21—H21	0.9300
C6—C7	1.369 (3)	C22—C26	1.364 (3)
C6—C14	1.373 (3)	C22—H22	0.9300
C7—H7	0.9300	C23—H23	0.9300
C8—C9	1.368 (2)	C24—C25	1.380 (3)
C10—C24	1.377 (2)	C24—H24	0.9300
C10—C18	1.378 (3)	C25—H25	0.9300
C10—C16	1.501 (3)	C26—C27	1.362 (3)
C11—C12	1.390 (2)	C26—H26	0.9300
C12—C14	1.381 (2)	C27—H27	0.9300
C12—H12	0.9300	C28—H28	0.9300
			
C2—N1—C8	105.97 (13)	C17—C15—C23	119.26 (17)
C2—N2—C9	106.82 (13)	C17—C15—H15	120.4
C2—N2—C16	128.26 (14)	C23—C15—H15	120.4
C9—N2—C16	124.44 (14)	N2—C16—C10	114.03 (14)
C7—C1—C11	121.22 (18)	N2—C16—H16A	107.8 (10)
C7—C1—H1	119.4	C10—C16—H16A	110.3 (10)
C11—C1—H1	119.4	N2—C16—H16B	108.6 (10)
N1—C2—N2	111.47 (14)	C10—C16—H16B	109.0 (10)
N1—C2—C11	123.73 (15)	H16A—C16—H16B	106.7 (13)
N2—C2—C11	124.79 (14)	C15—C17—C3	120.22 (17)
C17—C3—C4	121.31 (16)	C15—C17—H17	119.9
C17—C3—H3	119.3	C3—C17—H17	119.9
C4—C3—H3	119.3	C10—C18—C28	120.7 (2)
C3—C4—C20	117.33 (16)	C10—C18—H18	119.7
C3—C4—C8	119.95 (15)	C28—C18—H18	119.7
C20—C4—C8	122.61 (15)	C28—C19—C25	120.0 (2)
C21—C5—C13	118.12 (17)	C28—C19—H19	120.0
C21—C5—C9	122.19 (16)	C25—C19—H19	120.0
C13—C5—C9	119.68 (15)	C23—C20—C4	121.03 (17)
C7—C6—C14	121.57 (17)	C23—C20—H20	119.5
C7—C6—Cl1	119.75 (15)	C4—C20—H20	119.5
C14—C6—Cl1	118.69 (16)	C5—C21—C22	120.6 (2)
C6—C7—C1	118.92 (17)	C5—C21—H21	119.7
C6—C7—H7	120.5	C22—C21—H21	119.7
C1—C7—H7	120.5	C26—C22—C21	120.6 (2)
C9—C8—N1	109.98 (14)	C26—C22—H22	119.7
C9—C8—C4	129.88 (15)	C21—C22—H22	119.7
N1—C8—C4	120.14 (14)	C20—C23—C15	120.83 (18)
C8—C9—N2	105.76 (14)	C20—C23—H23	119.6
C8—C9—C5	132.19 (15)	C15—C23—H23	119.6
N2—C9—C5	121.91 (14)	C10—C24—C25	120.4 (2)
C24—C10—C18	118.34 (19)	C10—C24—H24	119.8
C24—C10—C16	122.53 (16)	C25—C24—H24	119.8
C18—C10—C16	119.12 (17)	C19—C25—C24	120.3 (2)
C1—C11—C12	118.42 (16)	C19—C25—H25	119.8
C1—C11—C2	122.62 (16)	C24—C25—H25	119.8
C12—C11—C2	118.87 (15)	C27—C26—C22	119.51 (19)
C14—C12—C11	120.65 (17)	C27—C26—H26	120.2
C14—C12—H12	119.7	C22—C26—H26	120.2
C11—C12—H12	119.7	C26—C27—C13	120.5 (2)
C27—C13—C5	120.73 (19)	C26—C27—H27	119.8
C27—C13—H13	119.6	C13—C27—H27	119.8
C5—C13—H13	119.6	C19—C28—C18	120.2 (2)
C6—C14—C12	119.22 (18)	C19—C28—H28	119.9
C6—C14—H14	120.4	C18—C28—H28	119.9
C12—C14—H14	120.4		
			
C8—N1—C2—N2	0.58 (18)	N1—C2—C11—C12	−39.5 (2)
C8—N1—C2—C11	−178.04 (15)	N2—C2—C11—C12	142.03 (17)
C9—N2—C2—N1	−0.62 (19)	C1—C11—C12—C14	0.9 (2)
C16—N2—C2—N1	171.59 (15)	C2—C11—C12—C14	177.51 (15)
C9—N2—C2—C11	177.97 (15)	C21—C5—C13—C27	−1.1 (3)
C16—N2—C2—C11	−9.8 (3)	C9—C5—C13—C27	177.59 (18)
C17—C3—C4—C20	−1.0 (3)	C7—C6—C14—C12	0.0 (3)
C17—C3—C4—C8	175.34 (17)	Cl1—C6—C14—C12	179.73 (13)
C14—C6—C7—C1	0.3 (3)	C11—C12—C14—C6	−0.6 (3)
Cl1—C6—C7—C1	−179.45 (12)	C2—N2—C16—C10	115.85 (19)
C11—C1—C7—C6	0.1 (2)	C9—N2—C16—C10	−73.2 (2)
C2—N1—C8—C9	−0.31 (18)	C24—C10—C16—N2	−28.4 (2)
C2—N1—C8—C4	−179.85 (15)	C18—C10—C16—N2	152.54 (16)
C3—C4—C8—C9	165.88 (18)	C23—C15—C17—C3	0.8 (3)
C20—C4—C8—C9	−17.9 (3)	C4—C3—C17—C15	0.3 (3)
C3—C4—C8—N1	−14.7 (2)	C24—C10—C18—C28	−0.3 (3)
C20—C4—C8—N1	161.51 (16)	C16—C10—C18—C28	178.75 (18)
N1—C8—C9—N2	−0.06 (18)	C3—C4—C20—C23	0.7 (3)
C4—C8—C9—N2	179.42 (16)	C8—C4—C20—C23	−175.60 (17)
N1—C8—C9—C5	175.61 (17)	C13—C5—C21—C22	0.9 (3)
C4—C8—C9—C5	−4.9 (3)	C9—C5—C21—C22	−177.70 (18)
C2—N2—C9—C8	0.40 (18)	C5—C21—C22—C26	0.1 (3)
C16—N2—C9—C8	−172.20 (15)	C4—C20—C23—C15	0.4 (3)
C2—N2—C9—C5	−175.82 (15)	C17—C15—C23—C20	−1.2 (3)
C16—N2—C9—C5	11.6 (2)	C18—C10—C24—C25	0.1 (3)
C21—C5—C9—C8	105.1 (2)	C16—C10—C24—C25	−178.92 (18)
C13—C5—C9—C8	−73.6 (3)	C28—C19—C25—C24	−0.1 (3)
C21—C5—C9—N2	−79.8 (2)	C10—C24—C25—C19	0.1 (3)
C13—C5—C9—N2	101.5 (2)	C21—C22—C26—C27	−1.1 (3)
C7—C1—C11—C12	−0.7 (2)	C22—C26—C27—C13	0.9 (3)
C7—C1—C11—C2	−177.10 (14)	C5—C13—C27—C26	0.2 (3)
N1—C2—C11—C1	136.88 (17)	C25—C19—C28—C18	−0.1 (4)
N2—C2—C11—C1	−41.5 (2)	C10—C18—C28—C19	0.3 (3)

### Hydrogen-bond geometry (Å, °)

**Table:**

*D*—H···*A*	*D*—H	H···*A*	*D*···*A*	*D*—H···*A*
C3—H3···N1	0.93	2.56	2.874 (2)	100
